# Estimating power in (generalized) linear mixed models: An open introduction and tutorial in R

**DOI:** 10.3758/s13428-021-01546-0

**Published:** 2021-05-05

**Authors:** Leah Kumle, Melissa L.-H. Võ, Dejan Draschkow

**Affiliations:** 1 Department of Psychology, Scene Grammar Lab, Goethe University Frankfurt, Frankfurt am Main, Germany; 2 Oxford Centre for Human Brain Activity, Wellcome Centre for Integrative Neuroimaging, Department of Psychiatry, University of Oxford, Oxford, UK

**Keywords:** Power, Mixed models, Simulation, lme4, mixedpower, R

## Abstract

Mixed-effects models are a powerful tool for modeling fixed and random effects simultaneously, but do not offer a feasible analytic solution for estimating the probability that a test correctly rejects the null hypothesis. Being able to estimate this probability, however, is critical for sample size planning, as power is closely linked to the reliability and replicability of empirical findings. A flexible and very intuitive alternative to *analytic* power solutions are *simulation-based* power analyses. Although various tools for conducting simulation-based power analyses for mixed-effects models are available, there is lack of guidance on how to appropriately use them. In this tutorial, we discuss how to estimate power for mixed-effects models in different use cases: first, how to use models that were fit on available (e.g. published) data to determine sample size; second, how to determine the number of stimuli required for sufficient power; and finally, how to conduct sample size planning without available data. Our examples cover both linear and generalized linear models and we provide code and resources for performing simulation-based power analyses on openly accessible data sets. The present work therefore helps researchers to navigate sound research design when using mixed-effects models, by summarizing resources, collating available knowledge, providing solutions and tools, and applying them to real-world problems in sample sizing planning when sophisticated analysis procedures like mixed-effects models are outlined as inferential procedures.

## Introduction

Linear mixed-effects models (LMMs), as well as generalized linear mixed models (GLMMs), are a popular and powerful choice in cognitive research, as they allow between-subject and between-item variance to be estimated simultaneously (for a discussion see Baayen, Davidson, & Bates, 2008; Kliegl, Wei, Dambacher, Yan, & Zhou, 2011). Moreover, (G)LMMs offer flexibility in dealing with missing data and unbalanced designs, and allow for a unified treatment of continuous and categorical responses (Baayen et al., 2008). There are plenty of excellent resources aimed at using (G)LMMs (e.g. Baayen et al., 2008; Bates, Mächler, Bolker, & Walker, 2014; DeBruine & Barr, 2021; Goldstein, 2007; Harrison et al., 2018), but far less guidance is available for experimental psychologists who want to estimate power for (G)LMMs (Brysbaert & Stevens, 2018; Westfall, Kenny, & Judd, 2014). However, accounting for statistical power while planning experimental designs is important for the reliability and replicability of empirical findings and a critical step for the successful preregistration of studies.

In this tutorial, we will consider different scenarios for a simulation-based power analysis and provide examples on how to perform such an analysis. This paper is designed to meet the needs of researchers who have some experience with mixed-effects modeling, and thus does not discuss topics such as model selection or optimal random-effects structure (Barr, Levy, Scheepers, & Tily, 2013; Bates, Kliegl, Vasishth, & Baayen, 2015a). Consequently, all scenarios introduced in this tutorial assume that an optimal model has already been selected. Thus, our aim is to provide tools and resources for researchers to use and explore simulation-based procedures, empowering them to find solutions for their own specific use cases. To achieve this, we use real-world data sets, rather than simplified examples, hoping that these examples would cover a wide problem space.

### Why is power important?

Statistical power is defined as the probability of rejecting the null hypothesis in favor of an alternative hypothesis when the null hypothesis indeed is false (Johnson, Barry, Ferguson, & Müller, 2015; Maxwell, Kelley, & Rausch, 2008). It is expressed by (1 – β), where β is the type II error probability—in simple terms, “if there is an effect of a certain size, what is the probability that my study will detect it”. The level of statistical power may be determined by balancing the researcher’s goal with the effort/cost needed to further increase power (Brysbaert & Stevens, 2018; O’Brien & Castelloe, 2007); however, in cognitive sciences, “80%” is often used as a minimal value. Statistical power for any experimental design depends predominantly on sample size, number of items (or trials), effect size, measurement variability, and the number of comparisons being performed (Coppock, 2013; Gelman & Carlin, 2014) (for a more detailed introduction to statistical power see Cohen, 1988). From these factors, sample size or number of items (i.e. number of observations) can be manipulated most easily. Keeping in mind the superordinate goal of conducting adequately powered studies, a power analysis is a helpful tool for planning your sample size (Nakagawa & Foster, 2004), which justifies the need for appropriate tools and guidelines to conduct such power analyses when sophisticated analysis procedures like (G)LMMs are used as inferential procedures.

### Why is it difficult to estimate power for (G)LMMs?

While (G)LMMs offer many advantages over traditional analysis procedures (e.g. conventional linear models, ANOVAs), their use is not as straightforward and requires careful deliberation (Matuschek, Kliegl, Vasishth, Baayen, & Bates, 2017). In addition to the specification of main effects and their interactions (i.e. fixed effects), (G)LMMs allow for the specification of parameters associated with the variance and correlation of random factors (e.g. of subjects and items) (Matuschek et al., 2017). As noted earlier, the power of a specific design is also influenced by the variability of responses. Consequently, all factors that have an influence on the variability of responses need to be accounted for when estimating power (Westfall et al., 2014). Since (G)LMMs capture multiple sources of random variations (Westfall et al., 2014), suitable power analyses procedures need to appropriately account for this increased complexity. Therefore, the same aspects that lead to the advantages of (G)LMMs and make them a popular tool in cognitive research also lead to increased difficulties in model specification and the analysis of power.

Classical approaches to power analysis typically work with analytical formulas which lack the necessary flexibility (Johnson et al., 2015; Thomas & Juanes, 1996) to solve power estimations for (G)LMMs (Brysbaert & Stevens, 2018; Green & MacLeod, 2016; Thomas & Juanes, 1996). One notable exception is Westfall, Kenny, and Judd’s (2014) analytical solution to power for mixed models with crossed random effects (e.g. a sample of participants respond to a sample of stimuli) (Shinyapp: https://jakewestfall.shinyapps.io/crossedpower/). Although extremely useful in certain cases, this solution is only applicable when calculating power for models with one fixed effect with two levels (Brysbaert & Stevens, 2018), i.e. very simple models. As more complex models are often used in practice, and power estimates for more than one fixed effect per model therefore become more frequent, the need for a different approach for (G)LMM power analysis becomes apparent. A flexible and very intuitive alternative to analytic power solutions are simulation-based power analyses (Brysbaert & Stevens, 2018; Thomas & Juanes, 1996).

### How does simulation-based power estimation work?

**Fig. 1:**
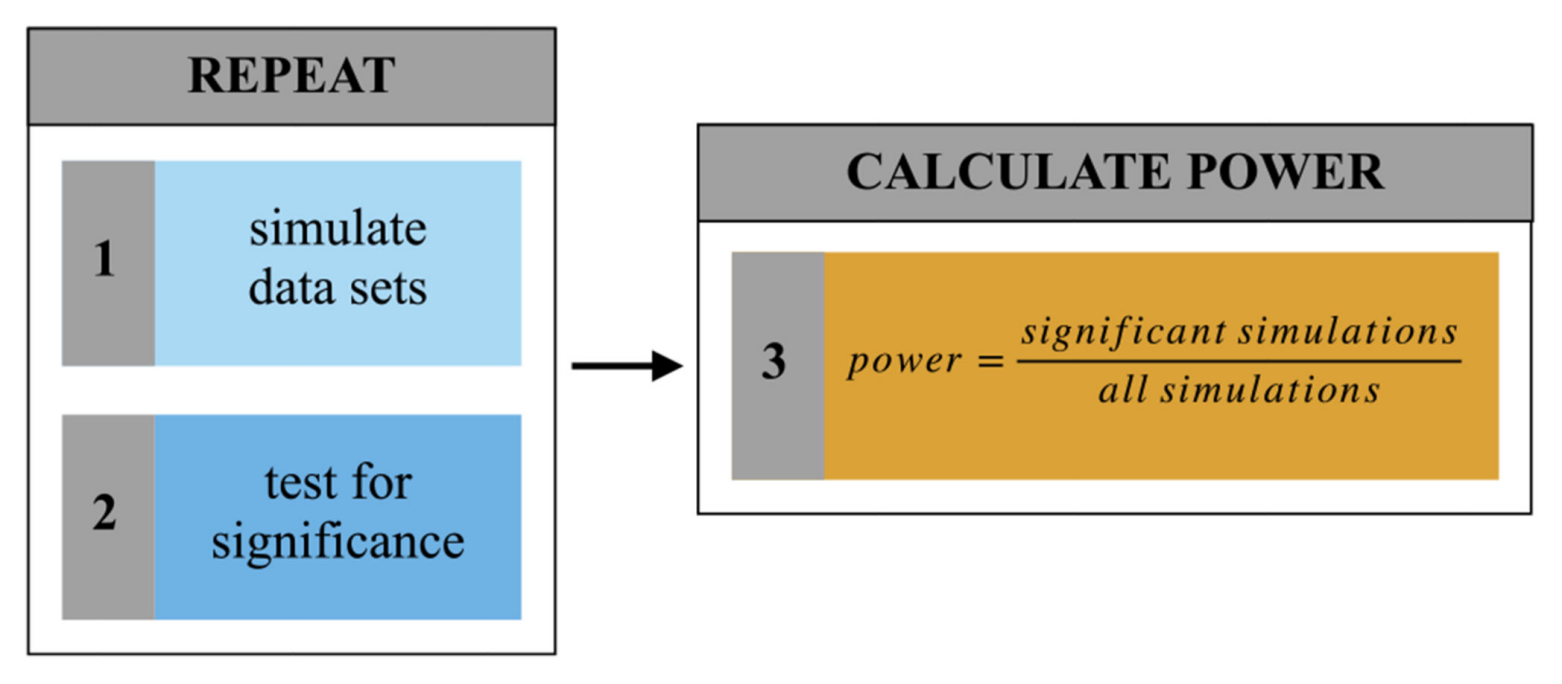
Basic principle behind a simulation-based approach to power analysis

In simple terms, one basic question behind power analyses is: “Suppose there really is an effect of a certain size and I run my experiment one hundred times-how many times will I get a statistically significant result?” (Coppock, 2013). As it is possible to simulate the outcome of an experiment, power can be calculated based on the proportion of significant simulations to all simulations (Johnson et al., 2015; Thomas & Juanes, 1996). The basic principle underlying all simulationbased power analysis solutions that we introduce in this paper can therefore be broken down into the following steps: (1) simulate new data sets, (2) analyze each data set and test for statistical significance, and (3) calculate the proportion of significant to all simulations (Fig. 1).

In principle, power can be estimated this way for most imaginable scenarios—as long as it is possible to simulate them. However, accuracy of the power estimate heavily depends on the accuracy of our simulation, since the probability of obtaining a significant result in a simulation is similar to the probability of the underlying real experiment only if the simulation is accurate (Thomas & Juanes, 1996). Informing the parameters of the simulation is therefore a critical step. As the simulations in the current situation need to be based on (G)LMMs, it is essential that we account for all of the relevant assumptions and parameters that enable the model to capture the underlying data structure correctly—that is, power estimation in (G)LMMs is not a trivial task.

In this tutorial, we will consider different scenarios and workflows for a simulation-based power analysis: (1) Scenario 1 focuses on using available well-powered data from previous experiments and how we may use models that were fit on such data to determine sample size; (2) Scenario 2 describes how to simulate power for more than one random variable (usually “participants”)—that is, determining the number of participants *and* stimuli required for sufficient power; and (3) in Scenario 3 the power analysis is based on self-generated artificial data in cases where no well-powered data are available. We will provide examples on how to perform these analyses on openly available data sets in the statistical programming language R, using the extremely popular *lme4* package (Bates, Mächler, Zurich, Bolker, & Walker, 2015b). The step-by-step procedures will be accessible as open Notebooks in the corresponding sections.

In all scenarios, we will estimate power in order to detect differences in the fixed effects (i.e. regression coefficients) of the model (but see e.g. Kain, Bolker, & McCoy, 2015, for detecting differences in the variation of random effects). Our aim is to provide researchers with an intuitive and practical understanding of simulation-based power analysis solutions for (G)LMMs. It is important to note that while this is a comprehensive resource, it is by no means an exhaustive one. Power analysis for mixed-effects models is still a largely uncharted terrain containing many open and unresolved issues. Thus, we highlight important pitfalls and considerations at every step.

### An important note applicable to all use cases

In general, models used to inform the simulation could originate from different data sources and could contain a range of model specifications, which would imply different requirements for a power analysis. Irrespective of the use case, it is imperative that the data and model used for simulation not stem from the experiment we want to estimate power *for*, but rather are *in-dependent from it* (Hoenig & Heisey, 2001). It should also be noted that the use of power analysis generally should be limited to planning studies and should not be used for analyzing or interpreting results (Hoenig & Heisey, 2001; Lenth, 2007). Since there is a monotonic mapping between post hoc power and *p*-values, computing post hoc power should not change the interpretation of *p*-values. Therefore, it is strongly recommended that power analyses not be performed once the results have been obtained (for a detailed discussion see Hoenig & Heisey, 2001).

## Scenario 1: Using an available well-powered design as a starting point

Basing the simulations on a preceding well-powered design is possibly the most desirable solution, since we can utilize a (G)LMM fitted on real and independent empirical data. This provides us with parameter estimates for fixed and random effects, as well as estimates for the coefficients of possible covariates, which eliminates guesswork and possibly biased assumptions. Here we will discuss the key steps as well as theoretical background for the problem-space of this scenario, whereas a step-by-step procedure can be found in Notebook 1. Our example focuses on a LMM with crossed random factors, but we also provide a notebook that demonstrates how to conduct a power analysis for a GLMM with nested random effects.

To more closely mimic real-world analysis demands, we intentionally demonstrate power estimation using a rather complex data set. We will work with data from a study published by Yan, Zhou, Shu, Yusupu, Miao, Kruegel, and Kliegl (2014) examining eye movements during reading. Yan et al. (2014) tested 48 subjects, each of whom read 120 sentences. During reading, gaze moves between different positions in the text to acquire all relevant information. Various factors can influence where a reader moves their eyes next. Amongst other questions, the authors investigated the effects of word length, word frequency, and morphological complexity (i.e. number of suffixes) on saccade’s first landing positions (FLP) during reading (i.e., the position in a word your eyes first land on). Suppose the goal is to conduct a study replicating and further investigating the effect of morphological complexity and word length on the saccades first landing position. In line with the results of Yan et al. (2014), we expect the FLP to increase (i.e. the eyes first land on a position further away from the start of the word) with increasing word length. However, we expect that morphological complexity interacts with this word length effect, such that more suffixes result in a FLP shift towards the beginning of the word. Here, we would need to conduct a power analysis in order to inform the sample size of the follow-up study.

**Fig. 2:**
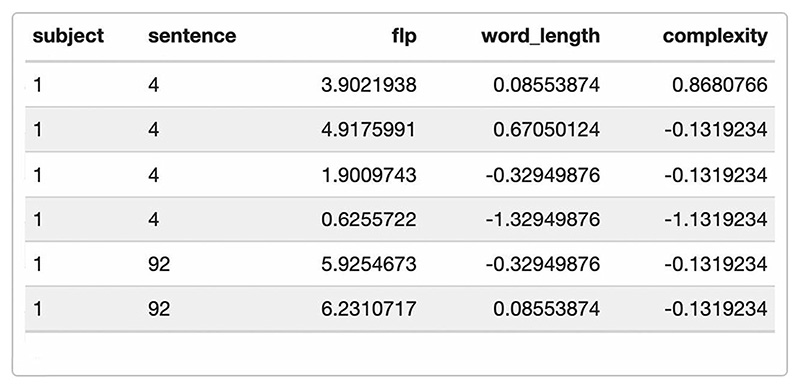
Data from Yan et al. (2014) including subject and sentence identifier (random factors) as well as the dependent variable “flp” (first landing position of saccades) and the predictors word length and complexity

First, we need the appropriate model fitted with lme4 and the data from Yan et al. (2014) available to us (Fig. 2). Note that all scenarios work under the assumption that an optimal model is selected prior to the power analysis (see e.g. Matuschek et al., 2017, for information on how power and model complexity interact).

To proceed, we use the preprocessed data frame of Yan et al. (2014) in which both continuous predictors are already centered. FLPmodel <— lmer(flp ~ word_**length** * complexity
                  + (1| subject) + (1| sentence), **data** = YanData)


The *FLPmodel* includes word length (β = 1.511) and morphological complexity (β = −0.075) as well as their interaction (β = 0.116) as fixed effects (see Table 1). Moreover, we included by-subject and by-item intercepts for the random variables *subjects* and *sentence*, making this model a typical example with crossed random effects as described by Baayen et al. (2008).

Generally, and with some amount of experience, it is possible to implement simulations from scratch. However, several premade software packages are available to simplify and speed up this process (e.g. *simglm* (LeBeau, 2019), *pamm* (Martin, 2012), *powerlmm* (Magnusson, 2018)). In this tutorial, we will focus on the two complementary packages *mixedpower* (Kumle, Võ, & Draschkow, 2018) and *simr* (Green & MacLeod, 2016), as they allow for power simulations for a wide range of (G)LMMs with different fixed- and random-effect structures.

### Mixedpower

As a start, *mixedpower* will be used to estimate power for the planned study (for a detailed introduction to all functions included in the package see the documentation). It allows for the estimation of power for all specified fixed effects and their interactions simultaneously and is comparatively time-efficient due to the parallelized nature of its computational architecture. While simulation-based power solutions for more complex models are still rather time-consuming, *mixedpower* is an efficient solution when power for multiple effects and parameter specifications is of interest—especially for large and complex data sets. We use *mixedpower* here since it is designed to be of didactic value and support intuitive understanding of simulation-based power estimation in general. For the sake of completeness, Notebook 1 additionally includes examples using the extremely flexible *simr* package (Green & MacLeod, 2016).

To determine the sample size for a prospective study, estimating power over a range of different sample sizes is highly informative. *Mixedpower* provides the eponymous *mixedpower*() -function which can be used to simulate power for one random variable (e.g. participants)—that is, the factor which is randomly sampled from the population we wish to generalize our results to. The simulation process inside *mixedpower*() closely follows the steps introduced in Fig. 1, with the first step consisting of simulating data sets. To achieve this, *mixedpower*() requires various pieces of information about the simulation process. 
*# simulate power for all fixed effects using mixedpower*
> **power**_FLP <— mixedpower (**model** = FLPmodel, **data** = YanData,
                           fixed-**effects** = **c**(“word-length”, “complexity”),
                           simvar = “subject”, steps = **c** (20, 30, 40, 50, 60),
                           critical_value = 2)


First, in addition to specifying the model and data, all fixed effects included in the model need to be stated explicitly. *Mixedpower* then uses the data entered and the structure captured by the fitted model to simulate new data using the simulate.merMod()-function in the *lme4* package (version > 1.1-6; Bates, Mächler, et al., 2015b). More specifically, simulate.merMod() is used to generate new values for the dependent variable from the provided, fitted model. Here, simulated values are sampled based on the distribution corresponding to the link function in the provided model (i.e. Gaussian distribution for LMMs or distributions corresponding to the “family” in GLMMs, e.g. “binomial”)—that is, the simulation process assumes that the dependent variable is following the distribution expected by the model type. Accordingly, the simulation of new values will be less appropriate if distributional assumptions are not met by the initial, fitted model. It is thus critical that the optimal model is selected prior to the power analysis.

Next, it is necessary to indicate which random variable should vary in the simulation (e.g. simvar = “subject”), which in this example implies that data sets with a range of different sample sizes are simulated in the power analysis procedure. *Mixedpower*() then creates a new data set containing simulated response values and the requested number of observations. Therefore, we will enter plausible sample sizes that we wish to estimate power for (e.g. steps of 20, 30, 40, 50, and 60). Subsequently, the simulated data are used to refit the model entered into the simulation and to perform an inferential significance evaluation (Fig. 1, step 2). The final parameter in need of specification in this simulation-based power framework, therefore, is the significance threshold. In general, increasing this threshold will lead to lower estimated power as it becomes harder to reach it, and vice versa. *Mixedpower* relies on *lme4*, which does not provide *p*-values. Even though there are methods available to compute *p*-values in mixed models, they come with ambiguity, because degrees of freedom in (G)LMMs are hard to determine (Baayen et al., 2008; Luke, 2017). *Mixedpower* therefore works with the available t-values for LMMs or z-values for GLMMs. All coefficients exceeding the selected t or z threshold value will be counted as significant. As it is plausible to have different criterions for different fixed effects (e.g. depending on whether the inclusion of an effect is of confirmatory or exploratory nature), *mixedpower* allows for the specification of different criterions for every effect as well as one criterion applied to all specified effects. For our use case we want to apply the same threshold to all specified effects, and thus will enter a t-value of 2 (critical_value = 2) into the simulation, as this will reflect an alpha level of 5% (Baayen et al., 2008). Additional details about the inner workings of *mixedpower* can be found in the documentation.

**Fig. 3:**
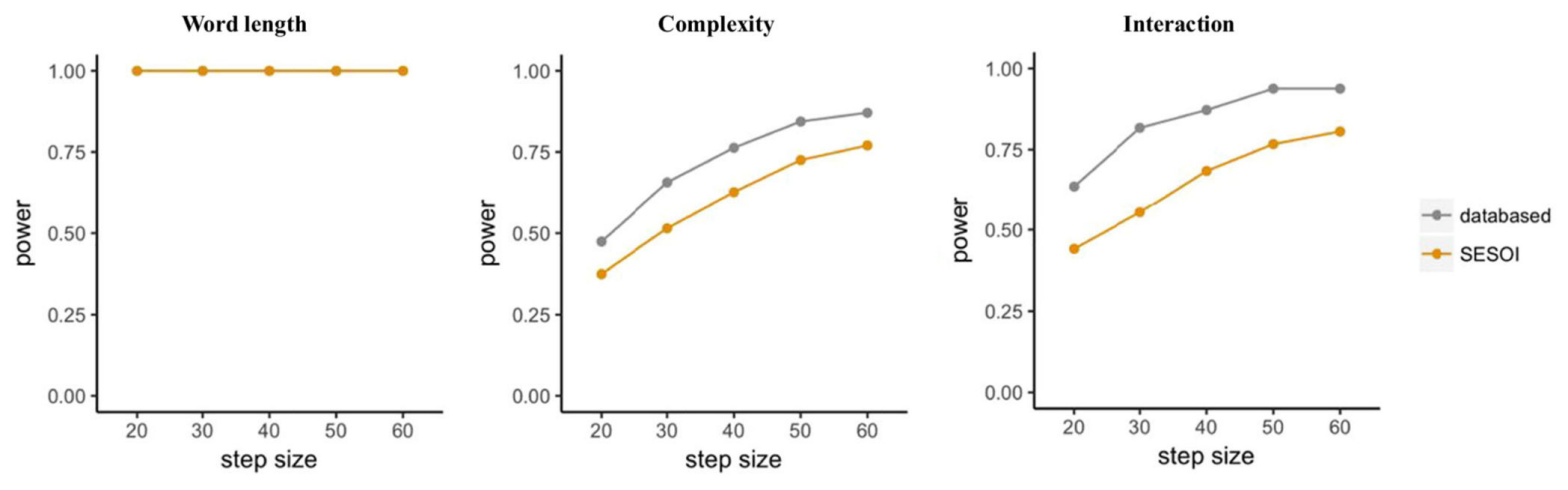
Results for power simulations in Scenario 1 using the *mixedpower*() function including power (y-axis) for the effect of word length (left panel), complexity (middle panel), and their interaction (right panel) as a function of the number of participants (x-axis). For each effect, data-based simulations (gray) as well as SESOI simulations (orange) are included

Figure 3 visualizes the outcome of the power analysis, with power increasing as sample size increases for one of the comparisons and the interaction. However, no changes in the effect of word length can be observed due to its large effect size. Since we used the exact coefficients (i.e. effect size) found in the empirical data, the corresponding results are data-based. Data-based estimations use the beta coefficients found in the empirical data, while SESOI (i.e. smallest effect of interest) estimations are based on adjusted effect sizes introduced below.

### Smallest effect size of interest

So far, all results rely entirely on the exact effect sizes found in the empirical data. Given the struggle for reproducibility in various subdomains of psychology (Ioannidis, 2005; Szucs & Ioannidis, 2017; Yong, 2012), adopting effect sizes from published data involves the risk of performing the analyses on inflated effect sizes, which in turn would result in an underpowered design. Therefore, a way of protecting against such bias or uncertainty in the data used for simulation is desirable. One approach is choosing *the smallest effect size of interest* (SESOI) to run a power analysis—making it possible to design studies which are worthwhile to run, as they have a predetermined power to detect an effect that is of interest (Albers & Lakens, 2018). This requires knowledge of what an effect “just large enough to be worth discovering” looks like and how to express it in the appropriate numerical scale of the model.

Determining the SESOI for (G)LMMs is difficult in a simulation-based approach where effect sizes are indicated through the model’s unstandardized beta coefficients. While Westfall et al. (2014) introduced a method for calculating effect sizes for designs with one fixed and two random effects (also see Judd, Westfall, & Kenny, 2017), relating effect sizes to beta coefficients in complex models is far from trivial, and the authors therefore refrain from making specific recommendations. Instead, we wish to highlight an approach introduced in Brysbaert and Stevens (2018). To change the effect size, the authors directly manipulated the data used to inform the power analysis (e.g. by adding a constant to the reaction time in a certain condition). Refitting the model with the manipulated data can then provide information on how such a change is reflected in the beta coefficients. However, we do acknowledge that this approach is likely not applicable in all use cases and that more work is needed to establish informed decisionmaking for SESOIs in (G)LMMs. Until then, guidance can come from previous research, literature, or practical constraints (for a more detailed discussion see Lakens, Scheel, & Isager, 2018). Additionally, repeating a power simulation for different plausible effect sizes that are not necessarily the SESOI is worthwhile, as this allows us to examine how sensitive a design’s power is to such changes, and to develop better intuition for the resulting power of different plausible scenarios.

Implementing this approach requires changing the beta coefficients in our model in order to run a SESOI power analysis. Coming back to the previous example and *FLPmodel*, SESOIs for the specified effects need to be selected and integrated into the model—allowing us to vary sample size and effect size simultaneously. The default values in *mixedpower*() (i.e. SESOI = False, databased = True), which we previously used when estimating “power_FLP” earlier, include the data-based (i.e. effects found in data) but not a SESOI simulation. To include a SESOI simulation, *mixedpower*() function can be handed a vector with SESOIs in the form of the desired beta coefficients for all specified fixed effects using the SESOI argument. Here, we default to a simple justification strategy of reducing all beta coefficients by 15%. Since we already computed a *data-based* simulation in “power_FLP”, we additionally set databased = False to make the next simulation as efficient as possible. 
*# define smallest effects of interest (also include Intercept)*
SESOI <- **c**(3.66, 1.3, -0.06, 0.09)
*# simulate power for different sample sizes and the specified SESOI*
**power**_SESOI <— mixedpower (**model** = FLPmodel_SESOI, **data** = YanData,
                           fixed-**effects** = **c**(“word-length”, “complexity”),
                           simvar = “subject”, steps = **c** (20, 30, 40, 50, 60),
                           critical.value = 2, SESOI = SESOI, databased = F)


As can be seen in Fig. 3, which combines the data-based simulation in “power_FLP” and the SESOI simulation in “power_SESOI”, simulations based on the SESOIs expectedly lead to more conservative estimates for the effect of complexity and the interaction between complexity and word length, while the main effect of word length is highly powered even for the specified SESOI and all examined sample sizes.

## Scenario 2: Simulating different units (random variables)

So far, we have demonstrated how to calculate power for varying effect sizes and varying sample sizes. Specifically, we manipulated the levels of one random variable (i.e. subjects) and the strength of our fixed effects (i.e. word length, complexity, and their interaction). The levels of the other random variable (i.e. stimuli/items), however, have been kept constant. Theoretically, it is possible to change the levels of this additional random variable in our design as well, which would influence the number of observations and thereby power (Brysbaert & Stevens, 2018). Scenario 2 covers situations where we wish to simulate power for different random variables/units (i.e. subjects or items) or different combinations of them. Step-by-step procedures and additional details concerning this scenario can be found in Notebook 2.

Returning to the previous example, another way to influence the power of our design is to change the numbers of sentences each subject is presented with. Using the *mixedpower*() -function, all that has to be changed compared to the previous simulation is the parameters simvar and steps, as we want to vary the number of sentences around the original number of 120 
*# simulate power for different number of sentences*
**power**_sentences <— mixedpower(**model** = FLPmodel, **data** = YanData,
                        fixed_**effects** = **c**(“word-length”, “complexity”),
                        simvar = “sentence”, steps = **c**(100, 120, 140,160, 180),
                        critical-value = 2, SESOI = SESOI)


**Fig. 4:**
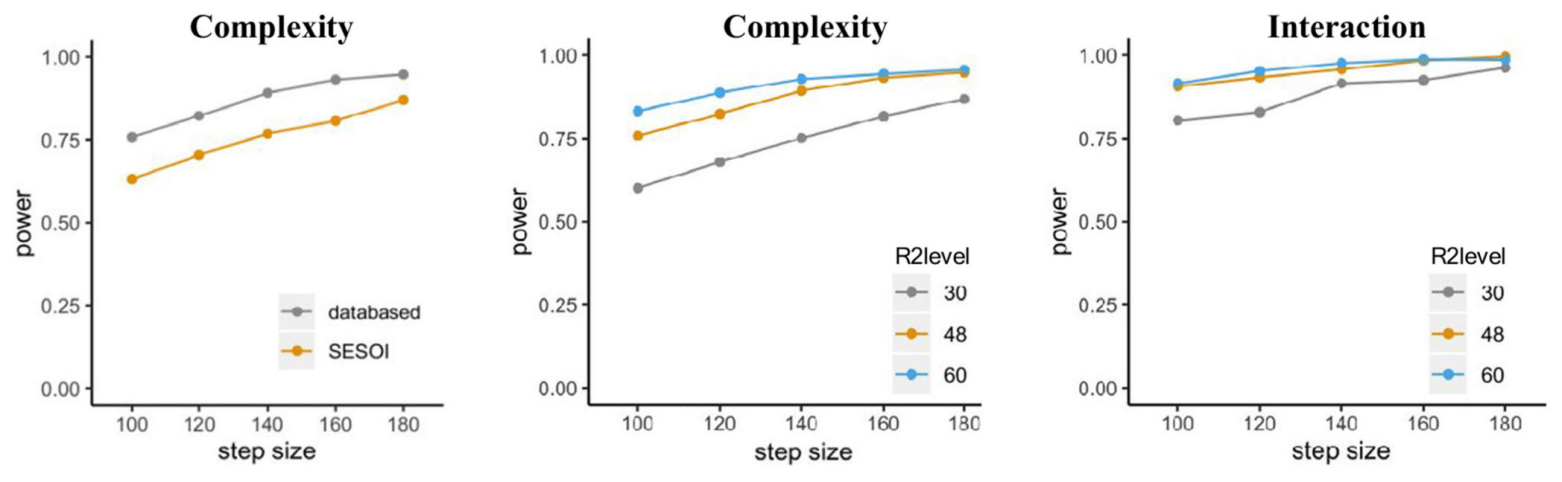
Left panel: Power for 48 participants for varying number of sentences (x-axis) including a data-based (gray) and SESOI (orange) simulation. Central panel: Data-based power for different sample sizes of participants (30, 48, 60) and varying numbers of sentences for the effect of complexity. Right panel: Data-based power for the interaction between complexity and word length

Concerns regarding the effect sizes used for simulating power hold true in this scenario as well; thus we will again include a simulation based on the specified SESOIs. Similarly to adding more subjects, including more sentences leads to higher power, and using more conservative effect size estimates leads to lower power (see Fig. 4, left panel, for results concerning the effect of “complexity” using the *mixedpower*() function pictured above).

However, being able to vary both simultaneously would be desirable, as it allows us to estimate power for different combinations of levels and to reduce the dependencies on the existing levels in the data used for simulation. To this end, *mixedpower* provides the function R2power(), which sets the levels of a specified random variable (e.g. number of sentences) while simultaneously changing the level of a second random variable (e.g. number of subjects).

Expanding upon the present example, suppose we now want to explore the effect of different numbers of sentences, but for 30 (or 60) subjects rather than the 48 subjects as in the original data set. Here, we choose the R2power() function in the *mixedpower* package, as it allows us to simulate sample sizes for one random variable (e.g. sentences) while simultaneously changing the level of an additional random variable (e.g. subjects). To implement this, the variable name of the additional random variable (R2var = “subject”) and its desired level (R2level = 30) need to be included in addition to the parameters specified in the *mixedpower*() function above. In a two-step simulation process, R2power() will first simulate a data set with the desired level of the simvar variable before simulating the chosen R2level. 
*# simulate power for 20 subjects (R2) and different numbers of sentences*
R2power <— R2power (**model** = FLPmodel, **data** = YanData,
                    fixed_**effects** = **c**(“word_length”, “complexity”),
                    simvar = “sentence”, steps = c(80, 100, 120, 140, 160),
                    R2var = “subject”, R2level = 30,
                    critical_value = 2)


R2power() closely mirrors the *mixedpower*() -function described in Scenario 1, with the only difference being the inclusion of an additional simulation step for each level of the variable varied in the steps argument (Fig. 1, step1), before determining statistical significance (Fig. 1, step 2). Repeating this process with different combinations of the set random effect (e.g. different R2 levels) enables an extensive overview of power and the factors that influence it. This can be especially useful in cases in which one of the random effects cannot be increased—for instance, some study-specific ceiling on the number of participants or stimuli. Setting the R2level to a number representing this limitation and simulating power for different R1 levels (steps) allows for the consideration of restrictions when deciding on a sample size.

In line with previous results in our example, more observations lead to higher power (Fig. 4, center and left panels). Even though not introduced in this scenario, R2power() also allows us to include a SESOI.

## Scenario 3: Having strong and detailed a priori assumptions

What if the simulation cannot be based on already existing data? In the scenario in which data or effect size estimates are either not available from previous research or the researcher already has substantiated expectations of the smallest effect size of interest, it is also possible to build data and model from scratch, thus bypassing the lack of appropriate existing data. Such custom-made model and data can then be used to inform the simulation and to estimate power for a study investigating the specified effects. However, as already noted, a priori simulated power is an accurate estimate only if the model used for simulation captures the underlying effects of interest well (Thomas & Juanes, 1996). Therefore, creating customized models that resemble the planned data and effect structure is a useful approach in cases where all parameters (e.g., fixed-effect coefficients, variance components associated with random effects) can be determined and justified a priori. Given the complicated nature of power analysis in (G)LMMs and the number of associated parameters discussed earlier, being able to justify each and all of them is not an easy task. Rather, substantial information regarding the expected data and model structure needs to be available. This method may become unsuitable for more complex models with a variety of fixed and random effects—as justifying and choosing parameters becomes more difficult with increasing model complexity.

Consequently, we will resort to a rather comprehensible example which is conceptually based on the *lexdec* data set in the *language R* package (Baayen, 2007). Parameters in the following analysis are therefore justified through this context. We will specifically focus on general approaches and considerations for parameter justification since we expect there to be much heterogeneity between different use cases. Here, it is difficult to define a best practice procedure, as parameters might need to be set without access to estimates from available data. The examples illustrated in this scenario therefore serve to introduce the general approach of setting up a power analysis from scratch while placing less focus on the exact parameter values. However, a detailed example of how to set up different artificial models and perform further power analyses can be found at in Notebook 3. Additionally, more extensive resources for data simulation in mixed models can be found in DeBruine and Barr (2021).

We consider the following situation: Researchers are planning a study investigating the effect of native language (English vs. non-English) in a lexical decision task where participants are asked to decide whether or not displayed letter strings form a word. The researchers expect native English speakers to be more accurate than non-native English speakers in discriminating English words from non-words. Besides native language, they are also interested in the effect of how common a displayed word is in the English language (i.e. word frequency) and hypothesize that more common words (e.g. chair) will be categorized more accurately than less common words (e.g. badger). Additionally, they want to investigate the interaction of word frequency and native language, since they expect that word frequency has a more profound influence on reading times for non-native English speakers. Accordingly, they plan to analyze their data with a GLMM for binomially distributed values, as the dependent variable of interest (i.e. correct classification: yes/no) is binary with two outcome possibilities. The predictors *native language* and *word frequency* are included in the model as fixed effects, together with bysubject and by-item (i.e. word) intercepts in the random-effect structure. The formula for such a model therefore is: 
**formula** <— Correct ~ NativeLanguage * Frequency + (1 | Subject) + (1 | Word)


At this stage the researchers want to calculate how many subjects and words they need to include in their study to achieve adequate power for their comparisons of interest, with particular emphasis being placed on the interaction term, since this constitutes the main question of the study. Critically, they have no available data and will thus need to create it first.

### Creating artificial data

To fit a GLMM with this formula, appropriate artificial data containing all important covariates are necessary. Starting with the random effects, variables identifying different subjects and words need to be created. We will be able to change the number of subjects and words later on in our power analysis, but for now will start with 20 subjects who each see 100 words. Using expand.grid() will lead to them being fully crossed. 
*# including variables used as random effects in artificial data*
artificial_**data** <–**expand.grid** (Word = (1:100), Subject = (1:20))


Moreover, variables coding the fixed effects native language and frequency are needed before determining appropriate effect sizes. We code the two levels of native language (English vs. non-English) as -0.5 vs. 0.5 (sum contrasts) and keep those two groups balanced. 
*# include native language*
artificial_**data** [“ NativeLanguage”] <— **c**(**rep**(—0.5, 1000), **rep**(0.5, 1000))


While coding the categorical predictor native language presents itself as fairly easy, more care needs to be given when simulating continuous predictors like *word frequency*. The underlying distribution can have a substantial effect on our power estimate, especially when the corresponding beta coefficient is kept constant rather than adjusted to the distribution and scale at hand. Ideally, a list of suitable words and their corresponding frequencies in English would have already been curated in order to use the actual frequency ratings in our artificial data, which would reduce the need for random sampling—a further source of variability in the simulation process. Here, we choose to sample word frequency from a normal distribution with a mean of 5 and a standard deviation of 1 (frequency ~ η(5,1)), as this distribution resembles the frequency ratings in the lexdec data set in the *language R* package (Baayen, 2007) and therefore can be justified using previous research (see Notebook 3). We subsequently center this continuous predictor. 
*# generate frequency ratings*
**frequency**_ratings <— **rnorm**(100, **mean** = 5, **sd** = 1)
*# repeat for every subject in data (20 times)*
artificial_**data** [“ Frequency”] <— **rep(frequency**. ratings, 20)
*# center variable*
artificial_**data$**CenteredFrequency <— **scale** (artificial_**data$**Frequency,
                                              **scale** = F)


**Fig. 5:**
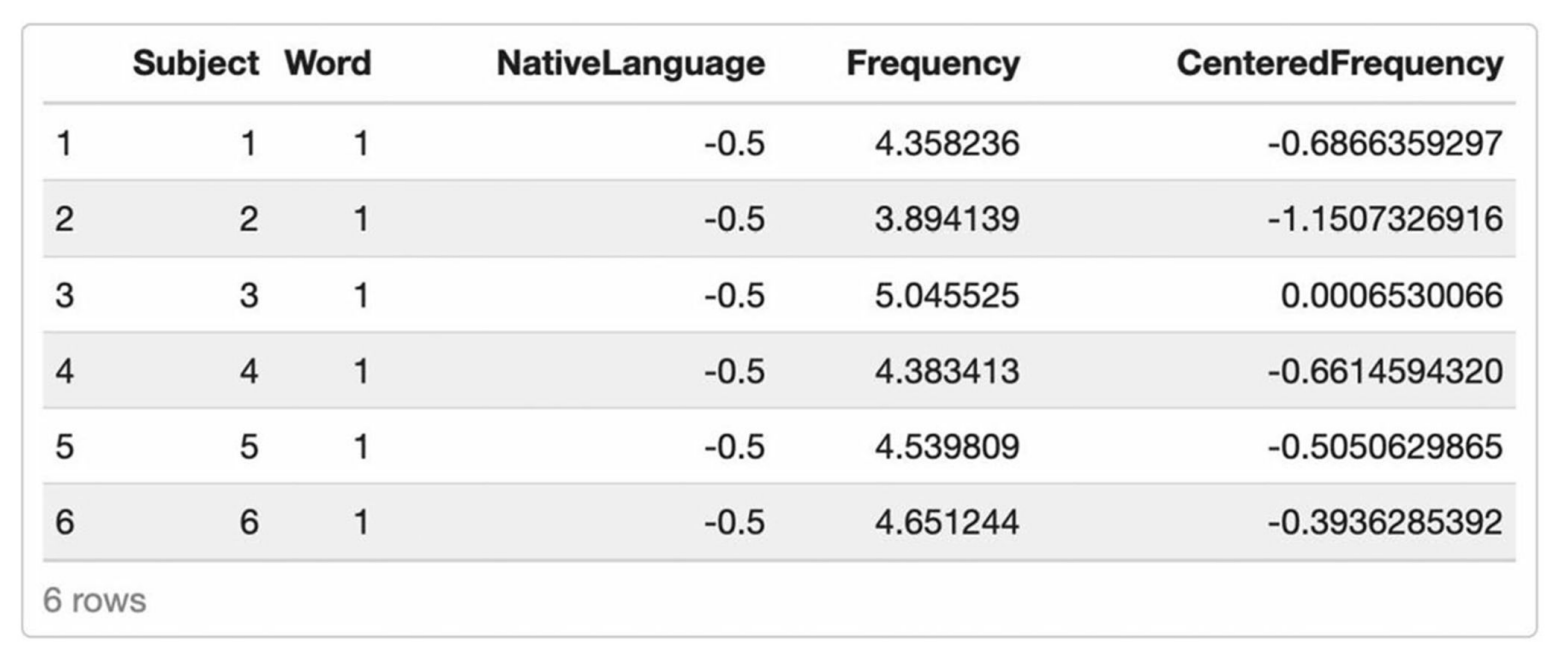
Artificial data frame created in Scenario 3, including all relevant predictors and covariates

Comparing the resulting artificial data frame (Fig. 5) with the intended model formula confirms that we now have included variables for all fixed and random effects.

As we still do not have values for our dependent variable, we cannot simply fit a model as we did in the previous scenarios. Different options exist for bypassing this dilemma, and we again want to highlight the resources provided in DeBruine and Barr (2019). We will make use of the *simr* package (Green & MacLeod, 2016), which includes functions to artificially create (G)LMMs with predefined beta coefficients and random variances. To do so, we need to specify the values for the fixed effects’ beta coefficients (i.e. effect sizes) and random effects’ variances. Values for those model parameters may come from the literature or the user’s own knowledge and experience.

However, choosing and justifying these parameters constitutes both the most critical and most difficult step in setting up a power analysis from scratch. First, trustworthy effect sizes, while having a substantial influence on power, are generally difficult to determine and justify. Second, while effect sizes are expressed in the form of beta coefficients, the mapping between them can be difficult. Mirroring the difficulties of determining SESOIs, researchers are therefore left with the extremely difficult and unintuitive task of specifying reliable beta coefficients.

Similar concerns apply to the justification of randomeffect variances. Since power in (G)LMMs also depends on the variation in its random variables, changes in this parameter can have a substantial effect on the model’s overall power, highlighting the fact that these parameter specifications need to be performed under extremely well-justified conditions (see Notebook 3 for simulations varying parameters in the random-effect structure). Note that while we work under the assumption that the final random-effect structure has already been selected, the complexity of the random-effect structure has been shown to have an influence on power as well—that is, power decreases with model complexity (Matuschek et al., 2017).

Undoubtedly, creating artificial data and models leaves open many decisions for researchers. On the one hand, this allows one to manipulate those parameters and to examine their effect on power; on the other hand, more parameters could lead to more potential misspecifications and require more extensive background knowledge to justify them. To make use of the advantages of this scenario as well as to protect against potential pitfalls, we strongly encourage users to simulate power for a range of plausible parameters concerning the artificial data in addition to the parameters modified in Scenarios 1 and 2. In the current example, we decided to take advantage of the flexibility introduced by creating data and model from scratch while at the same time using existing data as a basis of justification and knowledge. This essentially constitutes a hybrid of Scenarios 1 and 3. Since the hypothetical study in Scenario 3 closely mirrors the lexdec data (Baayen, 2007), we can fit a similar model to these data and use it to inform the beta coefficients and random variances of our artificial model (see Notebook 3). 
*# set values for intercept, fixed effects and interaction* 
fixed_**effects** <— **c**(—4.3, 0.35, —0.4, —0.32)
*# set random variances*
random.variance <— **list** (1.04, 0.65)


Having specified the fixed effects’ beta coefficients and random effects’ variances, we can make use of the makeGlmer() -function provided by *simr*, which combines all synthesized parameters and wraps them into an artificially fitted GLMM. For this to work, we need to provide the function with information about the distribution of the dependent variable (family = “binomial”). An example making use of the makeLmer() function in scenarios in which LMMs are planned can be found in Notebook 3. 
*# create artifical GLMM*
artificial_glmer <— makeGlmer (**formula**, fixef = fixed_**effects**,
                               VarCorr = random.variance,
                               **family** = “binomial”,
                               **data** = artificial_**data**)


Power analysis Once we have the same prerequisites as in Scenarios 1 and 2—namely data and a fitted model—we can continue with the actual power simulation and estimate power for a range of possible combination of parameters. Continuing to use simr, we can use the powerSim()-function to simulate power for exactly one specified fixed effect and the random-effects structure found in the model provided. 
*# analysis with simr for fixed effect “Native Language”*
**power**_simr <— powerSim(fit = artificial _glmer,
                       test = fixed (“NativeLanguage”), nsim = 1000)


As can be seen in Table 2, native language thus has a corresponding power of 16.7%.

To further explore power for the other fixed effects, we could repeat the above simulation for each predictor. However, once we have created an artificial model with simr, we can also take advantage of *mixedpower*’s faster runtime and flexibility to simulate power for all specified effects at the same time. To get a first overview of power for all predictors over a wide range of sample sizes, we will use the *mixedpower*() -function as in Scenario 1. 
*# power analysis with mixedpower for all fixed effects and interactions*
**power** <— mixedpower (**model** = artificial _glmer, **data** = artificial _**data**,
                     fixed_**effects** = **c** (“ NativeLanguage”, “ CenteredFrequency”),
                     simvar = “Subject”, steps = **c**(20, 60,100,140,180),
                     critical.value = 2,
                     SESOI = **c**(—4.3, 0.30, −0.34, −0.27))


**Fig. 6:**
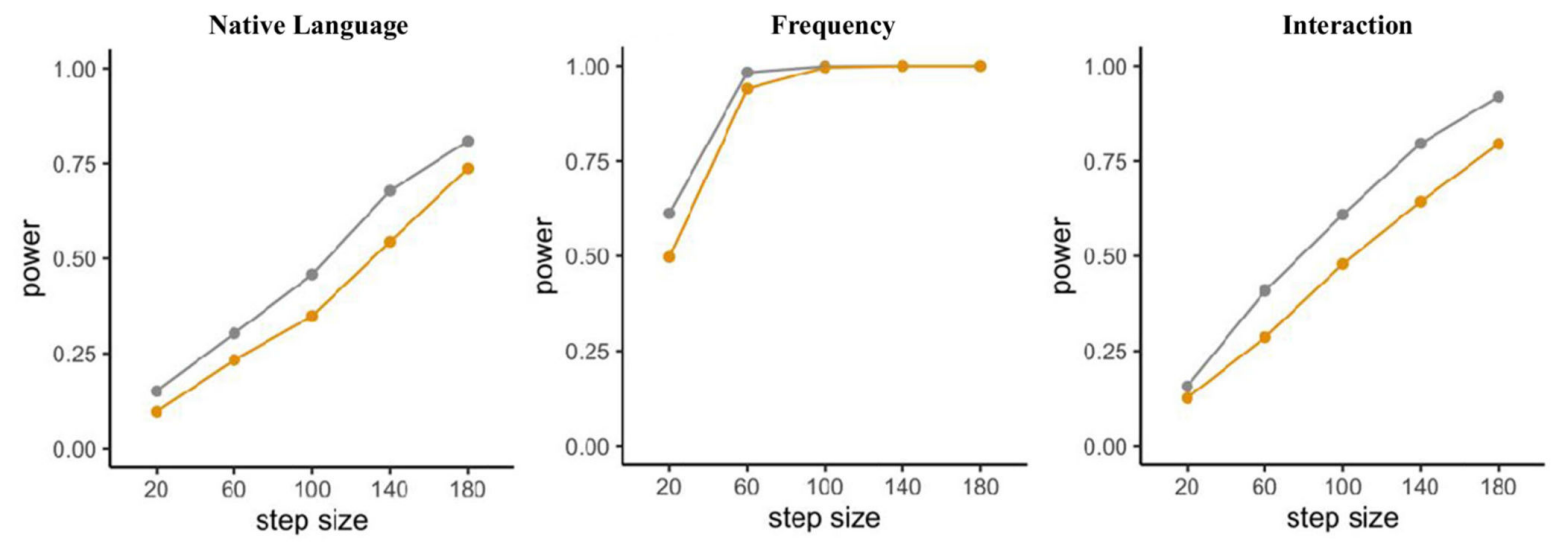
Power for the effects of Native Language, Frequency, and their interaction simulated with *mixedpower*

Moreover, we will again specify a different set of effect sizes through the SESOI argument to simultaneously inspect power for different effect sizes. Following the approach in Scenarios 1 and 2, we will reduce all beta coefficients by 15%. Results of the *mixedpower*() simulation can be seen in Fig. 6. Again, adding more subjects leads to higher power.

**Fig. 7:**
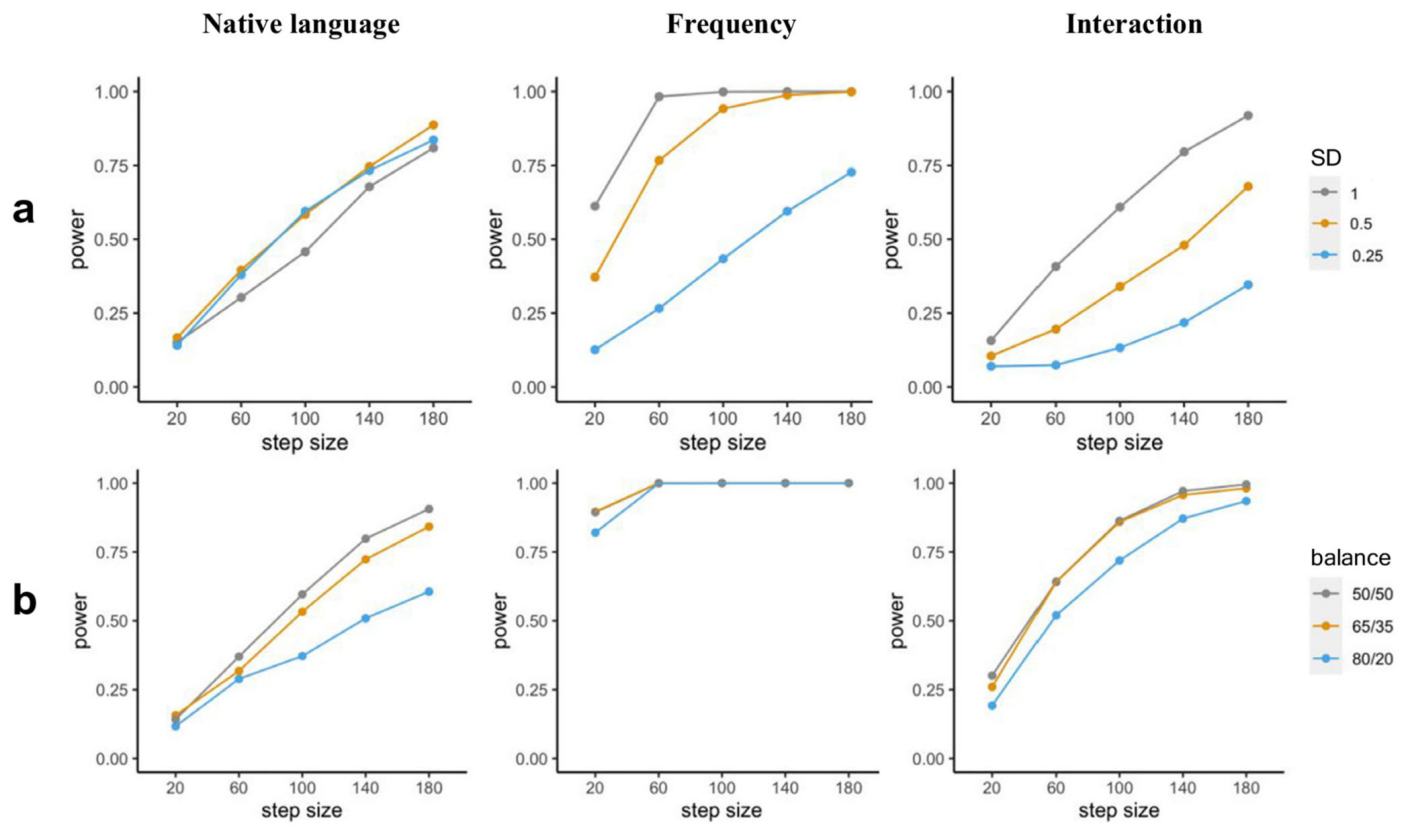
Power (y-axis) for all effects included in the artificial model for different assumptions made in the generation of the artificial data. **a** Power for different standard deviations in the distribution underlying the frequency ratings for the fixed effect “Frequency”. **b** Power for different ratios of English to non-English subjects (i.e. balance in variable native language)

Exploring parameters Since we custom-build the data and model used in the simulation, changes in parameters can be performed during this setup process and/or during parameter specification inside the functions provided by *mixedpower*. Thus, the workflow presented in this scenario enables researchers to explore how changing a range of parameters in the data creation and/or model specification influences power. Simulations can be especially helpful in exploring how the changes in different parameters influence the resulting inferences (DeBruine & Barr, 2019), and complementary tools like *mixedpower* and *simr* can extend this exploration into the domain of power analysis. As an example, we can manipulate the distributional properties of a continuous predictor (e.g. word frequency). Decreasing the standard deviation of the predictor to 0.5 can result in a substantial difference (up to 24%) in the power estimate (Fig. 7a), as it can have a profound influence on the effect size, illustrating again the need for appropriate justification of assumptions. We can also investigate how important balanced clusters in the variable *native language* (Fig. 7b) are. Thus far, we assumed that we will test as many native English speakers as non-native speakers. While researchers might have moderate control over which participants to recruit, it is possible to end up having unbalanced groups due to recruitment difficulties or limited access to special populations. Knowing the implications that unbalanced sampling might have for the power of a design *beforehand* can help in considering the impact of unbalanced groups while recruiting. Corresponding simulations can be found in Fig. 7b, suggesting that power can be lower for designs with unbalanced groups (Brysbaert, 2019; Konstantopoulos & Taylor, 2020). A final note on exploring parameters is that while we cover the option of changing the beta coefficients in a model, this method can also be used to simulate the type I error rate by setting all effect sizes to zero (Litière, Alonso, & Molenberghs, 2007).

## Interpretation of results

So far, the effect of different design parameters (e.g. sample size, number of items/stimuli, effect size) on power have been simulated in order to provide better intuition on how they influence a design’s statistical power. Additionally, we demonstrate that different additional parameters (e.g. balance across groups; Fig. 7b) can have a substantial influence on power. While power analyses are also a useful tool to illustrate these relationships, they mainly serve the purpose of determining and justifying design parameters (i.e. sample size or the number of items/stimuli) (Nakagawa & Foster, 2004). Being able to interpret and combine the results of power simulations of different effects of interest is therefore vital for a power analysis to meaningfully assist in experimental planning.

**Fig. 8:**
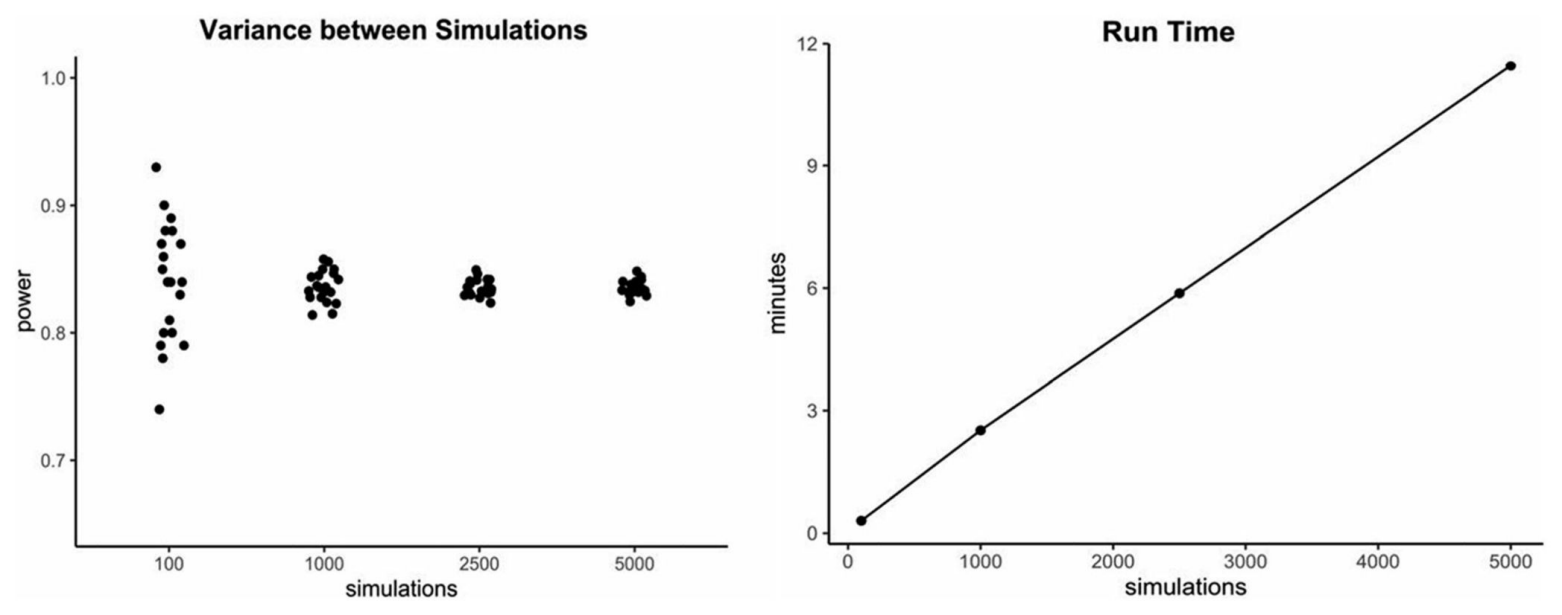
Variance (left) and mean runtime (right) for single power estimates and a different amount of underlying single repetitions/simulations. Here, power was estimated for the effect of complexity, for 48 subjects in the Yan et al. (2014) data set using *mixedpower*. Exact runtimes can vary extensively between different designs

First of all, it is important to note that the results of all power simulations are *estimations* and not exact calculations. Their accuracy is dependent on different factors, one of which is that a simulation needs to adequately reflect the underlying data structure (Thomas & Juanes, 1996). Since all mentioned power estimations are Monte Carlo simulations, which are an empirical method for evaluating statistics (Paxton, Curran, & Bollen, 2001), the results of a repeated simulation will almost always differ slightly. Keeping in mind that the last step of a simulation-based power analysis consists of calculating the proportion of significant to all simulations, we need to define how many iterations we want to perform. As a general rule, the greater the number of repetitions (i.e. single simulations) included in the simulation process, the more accurate and less affected by chance a result will be (Fig. 8, left). However, runtime increases as the number of repetitions included increases (Fig. 8, right), presenting a cost for efficiency.

We recommend running at least 1000 repetitions in the final simulation, which is the default value in all *mixedpower* functions. However, if runtime is not a constraint, more repetitions will lead to more accurate simulations and therefore are preferable (changes to the number of repetitions in *mixedpower* can be made over the nsim parameter). Moreover, recent advances in runtimes (e.g. the exceptionally fast Julia implementation of power simulations in the MixedModelSim package (GitHub repository) will allow for more efficient simulations, and therefore an easier inclusion of more repetitions in the future.

While it is important to keep in mind that power analyses only provide an estimate, the most important question still remains unanswered: How do we transform power curves to decisions about an experimental design? To begin with, we need to determine the level of power we are aiming for. Power of 80% is a common choice in experimental psychology (Brysbaert & Stevens, 2018); however, it is important to note that this does not reflect a fixed value, and different thresholds may be more appropriate depending on a researcher’s goal, or if an effect of interest is of exploratory or confirmatory nature. In this matter, different thresholds for different effects in the same (G)LMM can be appropriate. Secondly, we need to ensure that the simulations we run provide us with the information needed to make the decision, and we encourage researchers to simulate power for different combinations of relevant parameters to get a good overview of all factors. Most importantly, a strategy needs to be determined to combine the results for different effects and combinations after all relevant simulations are run. Preferably, parameters should be chosen that allow for meeting the power prerequisites for all effects of interest—that is, the most conservative parameters should be used to ensure sufficient power for all effects of interest (von Oertzen, 2010). However, different combinations of parameters (i.e. number of subjects or items/stimuli) might yield sufficient and equal overall power (i.e. *power equivalence*; von Oertzen, 2010; von Oertzen & Brandmaier, 2013), emphasizing the importance of finding the most appropriate and feasible combination. Guiding factors in this process can include available resources and practical constraints (e.g. a fixed number of stimuli that cannot be increased, limited funding). Decisions regarding the experimental design could therefore be motivated by reducing the overall cost or duration of the prospective study as well as ensuring that the study duration is manageable for a single participant (Kain, Bolker, & McCoy, 2015; von Oertzen, 2010)—all while ensuring sufficient overall power.

Returning to the example in Scenario 3, let us assume that we want to determine our sample size, and have already decided to include 100 words in the experiment: the results of our power analysis in Fig. 6 show large differences in power for the different effects. If we aim to achieve 80% power for all specified effects in the model, we would base our decision on the effect with the lowest overall power (i.e. native language), as this will result in an overall well-powered design. Using the data-based estimate, we would test 180 subjects. However, if the effect would be 15% smaller (as indicated by the SESOI estimate), even 180 subjects would not be enough to ensure adequate power.

Since all power simulations introduced in the present tutorial are based on estimating power for each effect separately, we recommend that sample size decisions be based on the smallest effect (i.e. with the lowest power) in the model. However, different strategies are available for combining power and determining significance in cases with complex fixed-effect structures (e.g. conditional power evaluations— that is, certain effect combinations to be significant *together* during the simulation process). Here, we want to highlight the option to specify different tests to determine significance in the *simr* package (see *simr* documentation for additional information) and believe this to be an important avenue for future work.

In all cases, these estimates only constitute the smallest sample size required to satisfy our goal (in this case 80%). As pointed out by Brysbaert (2019), running a few more participants than indicated by a power analysis comes with a minor financial cost but decreases the risk of ending up with an underpowered study.

Finally, it is important to report the results accordingly. In addition to the final decision for design parameters (e.g. sample size) and the corresponding power, all relevant parameters used in the power analysis should be reported. This includes effect sizes, if and which SESOI was used, and the level(s) of other random effects (e.g. number of items/stimuli).

## Tools and resources

There are various ways to conduct a power analysis for mixed models, with Westfall et al. (2014) and Brysbaert and Stevens (2018) constituting important introductory works in this domain. Here, we focus on the two packages *simr* (Green & MacLeod, 2016) and *mixedpower* (Kumle et al., 2018), which cover a wide range of possible use cases, and we encourage a complementary use of these tools (for a comparison of the two packages see Supplementary Notebook). Through parallel computing, *mixedpower* provides an efficient solution tailored to common scenarios in experimental and cognitive psychology, where complex models and designs with crossed random effects are prevalent. *Mixedpower* allows researchers to estimate, with relative efficiency, the power of fixed effects for different levels of random effects and thus can aid in decisions regarding sample size planning. Trading speed and efficiency against a wider range of use cases (e.g. setting up models from scratch, computing confidence intervals for power estimates), *simr* allows for more customizable simulations and therefore enables the user to exert more control over the simulation process; for instance, specifying the statistical test used to determine significance during the simulation process. However, we wish to acknowledge that additional useful software packages for simulation-based solutions to mixed models exist (e.g. *simglm* (LeBeau, 2019), *pamm* (Martin, 2012), *powerlmm* (Magnusson, 2018), *sim*. *glmm* (Johnson et al., 2015)), and that the resources introduced in this tutorial do not cover all available resources for power estimation in (G)LMMs.

## Conclusion

Considering power while planning experimental designs is important for the reliability and replicability of findings. To this end, a range of experimental parameters (e.g. sample size, number of stimuli/items) need to be justified and set accordingly for achieving adequately powered studies. However, power analyses are not necessarily a trivial task and may pose a feasibility barrier to researchers, especially when more sophisticated analyses like (G)LMMs are outlined for a study. In this tutorial paper, we provide code and resources to assist in the simulation-based computation of power, which should empower researchers not only to plan high-powered confirmatory studies but also to meet preregistration and submission requirements. We hope the tools and resources collated here will foster further exploration of simulation-based power analysis in (G)LMMs.

## Figures and Tables

**Table 1:** Summary of the FLP model

Random effects :			
Groups	Name	Variance	Std. Dev.
sentence	(Intercept)	0.05544	0.2355
subject	(Intercept)	0.35482	0.5957
Residual		3.20984	1.7916
Number of obs: 13523, groups: sentence, 120; subject, 48			
Fixed effects:			
	Estimate	Std. Error	t value
(Intercept)	3.66181	0.09081	40.323
word_length	1.51191	0.04605	32.832
complexity	–0.07534	0.02531	–2.977
word_length : complexity	0.11581	0.03293	3.517

**Table 2:** Results for the *simr* power analysis using powerSim()

Power for predictor ‘NativeLanguage’, (95% confidence interval) :
16.70% (14.44, 19.16)
Test : z–test
Effect size for NativeLanguage is 0.35
Based on 1000 simulations, (11 warnings, 0 errors)
alpha = 0.05, nrow = 2000
Time elapsed : 0 h 23 m 0 s

## Data Availability

The data sets analyzed in the current manuscript were derived from the following public domain resources: http://read.psych.uni-potsdam.de/index.php?option=com_content&view=article&id=132:yan-et-al-2014-cognition-eye-movements-guided-by-morphological-structure-evidence-from-the-uighur-language&catid=12:publications&Itemid=11
